# The origin of introns and their role in eukaryogenesis: a compromise solution to the introns-early versus introns-late debate?

**DOI:** 10.1186/1745-6150-1-22

**Published:** 2006-08-14

**Authors:** Eugene V Koonin

**Affiliations:** 1 National Center for Biotechnology Information, National Library of Medicine, National Institutes of Health, Bethesda, MD 20894, USA

## Abstract

**Background:**

Ever since the discovery of 'genes in pieces' and mRNA splicing in eukaryotes, origin and evolution of spliceosomal introns have been considered within the conceptual framework of the 'introns early' versus 'introns late' debate. The 'introns early' hypothesis, which is closely linked to the so-called exon theory of gene evolution, posits that protein-coding genes were interrupted by numerous introns even at the earliest stages of life's evolution and that introns played a major role in the origin of proteins by facilitating recombination of sequences coding for small protein/peptide modules. Under this scenario, the absence of spliceosomal introns in prokaryotes is considered to be a result of "genome streamlining". The 'introns late' hypothesis counters that spliceosomal introns emerged only in eukaryotes, and moreover, have been inserted into protein-coding genes continuously throughout the evolution of eukaryotes. Beyond the formal dilemma, the more substantial side of this debate has to do with possible roles of introns in the evolution of eukaryotes.

**Results:**

I argue that several lines of evidence now suggest a coherent solution to the introns-early versus introns-late debate, and the emerging picture of intron evolution integrates aspects of both views although, formally, there seems to be no support for the original version of introns-early. Firstly, there is growing evidence that spliceosomal introns evolved from group II self-splicing introns which are present, usually, in small numbers, in many bacteria, and probably, moved into the evolving eukaryotic genome from the α-proteobacterial progenitor of the mitochondria. Secondly, the concept of a primordial pool of 'virus-like' genetic elements implies that self-splicing introns are among the most ancient genetic entities. Thirdly, reconstructions of the ancestral state of eukaryotic genes suggest that the last common ancestor of extant eukaryotes had an intron-rich genome. Thus, it appears that ancestors of spliceosomal introns, indeed, have existed since the earliest stages of life's evolution, in a formal agreement with the introns-early scenario. However, there is no evidence that these ancient introns ever became widespread before the emergence of eukaryotes, hence, the central tenet of introns-early, the role of introns in early evolution of proteins, has no support. However, the demonstration that numerous introns invaded eukaryotic genes at the outset of eukaryotic evolution and that subsequent intron gain has been limited in many eukaryotic lineages implicates introns as an ancestral feature of eukaryotic genomes and refutes radical versions of introns-late. Perhaps, most importantly, I argue that the intron invasion triggered other pivotal events of eukaryogenesis, including the emergence of the spliceosome, the nucleus, the linear chromosomes, the telomerase, and the ubiquitin signaling system. This concept of eukaryogenesis, in a sense, revives some tenets of the exon hypothesis, by assigning to introns crucial roles in eukaryotic evolutionary innovation.

**Conclusion:**

The scenario of the origin and evolution of introns that is best compatible with the results of comparative genomics and theoretical considerations goes as follows: self-splicing introns since the earliest stages of life's evolution – numerous spliceosomal introns invading genes of the emerging eukaryote during eukaryogenesis – subsequent lineage-specific loss and gain of introns. The intron invasion, probably, spawned by the mitochondrial endosymbiont, might have critically contributed to the emergence of the principal features of the eukaryotic cell. This scenario combines aspects of the introns-early and introns-late views.

**Reviewers:**

this article was reviewed by W. Ford Doolittle, James Darnell (nominated by W. Ford Doolittle), William Martin, and Anthony Poole.

## Open peer review

Reviewed by W. Ford Doolittle, James Darnell (nominated by W. Ford Doolittle), William Martin, and Anthony Poole. For the full reviews, please go to the Reviewers' Comments section.

## Background

### 'Introns early' versus 'introns late'

The discovery, in 1977, of the discontinuous structure of eukaryotic genes and the splicing mechanisms that put pieces of genes (exons) together was, beyond doubt, not only one of the most fundamental but also one of the most unexpected and puzzling discoveries in the 20^th ^century biology [1,2]. Almost immediately, the key question 'Why genes in pieces?' has been posed by Walter Gilbert who introduced, all in the same seminal News & Views article, the terms 'exons' and 'introns', and postulated major evolutionary significance of split genes thanks to the potential of exon shuffling and alternative splicing [3]. Subsequently, these ideas have been consolidated in the 'exon theory' of gene evolution [4]. Immediately, Ford Doolittle offered a provocative retort: 'Were they ever together?" where he speculated that the split state was ancestral to genes despite the fact that introns are missing in prokaryotes (or so it appeared at the time) [5]; similar ideas have been developed almost simultaneously by James Darnell[6].

Thus the 'introns early' hypothesis (hereinafter introns-early) was born. This concept is obviously linked to the 'exon theory' of genes independently proposed by Gilbert and Blake – the two ideas converge on the notion that introns played a crucial role in the origin of proteins by facilitating recombination of protein modules [7-9]. More specifically, it has been postulated that the earliest genetic elements encoded small domains, conceivably, of the size close to the typical length of modern exons (~50 amino acids) which recombined via non-coding sequences (to become introns) present in some of these elements to yield 'genes in pieces' encoding full-sized proteins [10-12]. This attractive idea circumvents the need for the unlikely 'invention' of long open reading frames and seems to account for the characteristic domain organization of proteins. The central corollary of introns-early is that subsequent evolution of genes involved, mostly, loss of introns, partial in eukaryotes and complete in prokaryotes.

The absence of introns in prokaryotes which is, certainly, a potentially embarrassing complication, if not the mortal blow to introns-early, has been explained away by postulating the 'genome streamlining' mode of evolution for prokaryotes [4,12,13]. Under the streamlining hypothesis, the main pressure in the evolution of prokaryotes had been maximization of the replication rate, hence elimination of all non-essential parts of the genomes. The introns, obviously, would not survive under this evolutionary regime in the vast prokaryotic populations affected by intense purifying selection.

The alternative to introns-early is the 'introns late' scenario (hereinafter introns-late) under which introns are a eukaryotic innovation and, moreover, intron gain has been a continuous process during evolution of eukaryotes [14-18]. Introns-late is, more or less, a 'what you see is what you get' concept according to which prokaryotes – organisms that currently have no spliceosomal introns and, of course, no spliceosomes – have never had them in the first place.

In the years after the formulation of the original, 'strong' introns-early hypothesis, the discovery of numerous lineage-specific introns persuaded Gilbert and coworkers to modify their concept by incorporating aspects of introns-late [10,12,19]. The new version – introns-early in the age of genomics – allows for relatively late (presumably, at different stages of eukaryotic evolution) gain of a substantial fraction of introns but sticks to the central theme of introns early which is, essentially, the exon theory: that presence of many spliceosomal introns in protein-coding genes is an ancestral feature of genetic systems. Therefore, throughout the further discussion, I refer to this new, 'weaker' version as 'introns-early'.

Introns-early is a decidedly non-parsimonious scenario in postulating that a genomic feature that is absent in two of the three domains of life, bacteria and archaea, is nevertheless ancestral. Of course, parsimony is a statistical principle that does not necessarily apply to singular events in life's evolution. However, for a non-parsimonious scenario to be viable, it must make clear, readily falsifiable predictions. Introns-early (the exon theory) leads to at least four such predictions although only the first two have been extensively investigated by the proponents of this hypothesis: i) if primordial genes evolved by recombination of exon-size coding segments, there should be a substantial excess of phase '0' introns (inserted between codons) over phase '1' and '2' introns (inserted within a codon), ii) exon boundaries should correspond to domain/module boundaries in proteins, iii) ancient paralogs that evolved as a result of gene duplications antedating the advent of eukaryotes should share at least some intron positions, iv) there should be a difference in intron density and properties between eukaryotic genes of different age, e.g., between ancestral genes inherited from archaea and genes transferred from the mitochondrial endosymbiont.

At face value, the first prediction seems to hold – there is, indeed, a significant excess of phase 0 introns in all sequenced eukaryotic genomes[13,20-23]. However, more mundane explanations for this pattern, based on selection acting on splice junctions and the concept of protosplice sites (sites of preferential intron insertion), have been proposed. In particular, the excess of phase 0 introns has been attributed to preferential fixation of inserted introns in front of a synonymous position (3^rd ^base of a codon) allowing selection for splicing efficiency [24]. Moreover, contrary to the prediction of the introns-early hypothesis, the excess of phase 0 is somewhat greater among 'new' introns that appear to have been inserted into genes relatively late in eukaryotic evolution, as opposed to 'old' introns that might comprise the primordial heritage under introns-early (see below)[23].

The second prediction, the correspondence between exons and protein domains (modules), is the one that has been tested most extensively, and the results have been cited in support of introns-early[10,25-27]. However, the distribution of exon lengths alone already indicates that there can be no straightforward exon-domain correspondence. Indeed, the mean size of an exon in most species is ~50 codons whereas an average domain is about twice as large. Attempts have been made to define structural modules in proteins in such a manner that they would correspond to exons [25,26] but, despite claims of success, the consensus remains that there is no objective support for such a correspondence [14,15,17,28-31]. There seems to be a role for exon shuffling in the combinatorial evolution of animal multidomain, extracellular proteins but this is, obviously, a late, animal-specific development that cannot provide any support for introns-early [32-34].

The prediction on conservation of intron positions in ancient paralogs, those that predate the Last Universal Common Ancestor (LUCA) of the modern life forms, has been formulated and tested, albeit on a limited data set, in an elegant study of Cho and Doolittle [35]. The results were clear-cut: no trace of intron position conservation, hence no support for introns-early.

The test for intron density and phase distribution in genes of different ages and origins has been proposed and performed, albeit in a crude manner, by Wolf et al[36]. In this work, the genes in the nematode genome were partitioned into those with closest bacterial homologs, hence, probably, of relatively late, conceivably, mitochondrial origin, and those with closest archaeal homologs and more distant homologs in bacteria, i.e., probably, inherited from LUCA. A comparison of intron density and properties yielded almost paradoxical results: if anything, the "young" genes had a slightly higher intron density and a slightly greater content of phase 0 introns. A subsequent, conceptually similar comparison of introns whose position is conserved in distant eukaryotic lineages (e.g., plants and animals) – old introns, and lineage-specific, young introns in a carefully curated gene set produced nearly identical results[23].

More recently, the two approaches have been combined in a comparative analysis of intron positions in genes for cytosolic and mitochondrial ribosomal proteins [37]. These genes are ancient paralogs, inasmuch as the cytosolic ribosomal protein genes derive from the archaeal ancestors whereas those for mitochondrial ribosomal proteins are of bacterial origin, but they also have been inherited by eukaryotes via different routes, i.e., from the archaeal host and the mitochondrial endosymbiont, respectively. The two groups of genes have been found to contain introns, largely, in different positions, with the few coincident ones being well within the expected number of parallel gains. Thus, introns seem to have inserted independently into these ancient paralogs, contrary to the introns-early prediction.

Taken together, these independent lines of evidence seem to refute introns-early, even in its modified form, which allows some late insertion of introns: there is no indication that the genes of LUCA contained numerous introns, that prokaryotes underwent genome streamlining, or that exon shuffling had any role in the emergence of the first genes. However, the introns-early hypothesis incorporated too many good ideas to just go out with a whimper; in the rest of this article, we shall see that things are not quite easy for introns-late either and that the latest results of comparative genomics converge with general, conceptual thinking to stage a partial, modest but tangible renaissance of "introns-early".

## Results and discussion

### Reconstruction of evolution of eukaryotic genes: numerous introns from the outset of eukaryotic evolution

Sequences of multiple, complete genomes of eukaryotes from various lineages enabled reconstruction of ancestral gene structures and of evolutionary trajectories that led to the modern genes [38]. In essence, the sequences of a set of orthologous genes are aligned, the positions of introns are mapped, and the mapping is converted into a 0–1 matrix. This matrix can then be treated with any of a variety of available methods and models for intron gain and loss to reconstruct the corresponding evolutionary scenario. Of course, any scenario thus constructed depends on the topology of the underlying phylogenetic tree, and this is far from being resolved in the case of eukaryotes. The simplest approach is evolutionary parsimony which deterministically assumes the scenario with the smallest number of events (in this case, intron gain and loss) to represent what most likely happened in evolution[23,39]. An important limitation of this approach is that it penalizes multiple losses of introns and, accordingly, is likely to underestimate the number of ancestral introns, perhaps, substantially, if loss of an intron in the same position in different lineages is common. More sophisticated methods for reconstruction of evolutionary scenarios are, mostly, different maximum likelihood (ML) and mixed parsimony/ML models [40-43]. These models include some finite likelihood that unique modern introns are actually ancestral, due to losses in multiple lineages, and so have the potential of obtaining more accurate estimates of the number of ancestral introns. However, ML models also have many parameters that are hard to define optimally, hence the potential for error, in particular, overestimate of ancestral introns.

**Figure 1:**
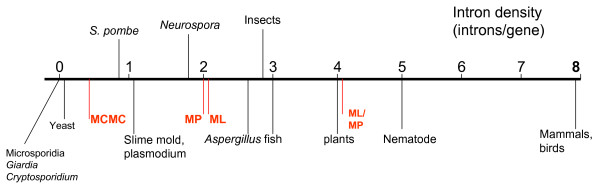
**Intron density in the genes of some modern eukaryotes and the reconstructed common ancestor of plants and animal (possible LECA under unicont-bicont phylogeny)**. The marks on the line show approximate intron density values (mean number of introns per gene); the data is from [99]. The reconstructed values for the ancestral genome (in red) are denoted after the reconstruction method: MCMC, Markov Chain Monte Carlo[49]; MP, maximum parsimony[23]; ML, maximum likelihood[41]; ML/MP, hybrid maximum likelihood/parsimony method[51].

The quantitative differences notwithstanding, the parsimony and ML reconstructions yield qualitatively agreeing results: the common ancestor of animals and plants had a surprisingly intron-rich genome (Fig. 1). Depending on the eukaryotic tree topology, this might mean that the Last Eukaryotic Common Ancestor (LECA) also was quite intron-rich – indeed, according to the unikont-bikont phylogeny that has recently come into vogue, the last common ancestor of animals and plants was the same as LECA [44-46]. Even under more traditional eukaryotic tree topologies, where some of the unicellular eukaryotes are taken to be early-branching lineages. [47,48], the possibility that LECA was intron-rich looms large because many unicellular forms easily could have lost most of the introns. In certain cases, where the tree topology was unambiguous, in particular, in yeast *Saccharomyces *and in microsporidia, this eukaryotic genome streamlining is undeniable[23].

Apart from the qualitative similarity of the conclusions, the parsimony and ML approaches substantially differ in the estimates of the relative extent of intron gain and loss in eukaryotic evolution. The hybrid parsimony/ML model of Roy and Gilbert yielded an extreme picture of eukaryotic gene evolution that is strongly dominated by intron loss[40]. Essentially, the history of eukaryotic genes according to Roy and Gilbert amounts to appearance of (nearly) all introns in LECA, with (almost) all of the subsequent evolution limited to lineage-specific gene loss [13]. In a sharp contrast, a different type of probabilistic model, Markov Chain Monte Carlo, offers an extreme introns-late scenario, with (nearly) no introns having survived from LECA [49]. The parsimony reconstruction as well as more general and refined ML models [41-43], suggest a mixed scenario whereby at least some of the eukaryotic lineages, in particular, animals and plants, have gained a substantial number of introns, actually, more than the common ancestor of plants and animals is inferred to have had (Fig. 1). On the weight of all evidence, and despite the great uncertainty that is associated with the inference of intron density in the root of the tree (LECA) under ML models, these mixed scenarios seem more likely than those dominated by intron loss or intron gain [38]. However, mechanisms of intron insertion remain a mystery, and furthermore, it seems that intron gain, if it, indeed, occurred repeatedly during eukaryotic evolution, has been episodic and, perhaps, associated with major evolutionary transitions, e.g., origin of animals, as opposed to the more uniform (even if lineage-specific) intron loss process[50,51]. Indeed, it appears certain that, for example, during the evolution of mammals (~100 million years), and probably, during the evolution of vertebrates (~600 million years), there has been virtually no intron gain [50,52]. Other eukaryotic lineages might have a higher intron gain rate, though, as illustrated by evidence of apparent recent gain in nematodes [53].

Regardless of the details of evolutionary reconstructions, comparative genomics has compellingly shown that numerous introns have resided in eukaryotic genomes since very early in eukaryotic evolution if not from its very outset. This conclusion, of course, does not redeem the original introns-early idea which requires numerous introns at the earliest stages of life's evolution not just at the origin of eukaryotes. However, it does go a step in that direction: at least, the radical introns-late position, i. e., the scenario of continuous intron insertion during eukaryotic evolution, such that most, if not all, introns are relatively new, is hardly defendable anymore. To gain further insight into the status of introns-early, we must now turn to the ultimate origin of spliceosomal introns.

### The apparent origin of spliceosomal introns and eukaryotic retroelements from group II self-splicing introns

Although, when introns have been discovered, their origin appeared completely mysterious, the discovery of self-splicing introns offered a solution that appears increasingly plausible as the diversity of these elements is being explored in its fascinating details [54,55]. The likely ancestors of spliceosomal introns are the group II self-splicing introns whose terminal structures, which form the splicing ribozyme, show remarkable similarity to the structures of the spliceosomal snRNAs and, especially, the complexes formed between snRNAs and the ends of spliceosomal introns [54-56]. It appears that, during the early evolution of eukaryotes, group II introns fragmented into the active, spliceosomal part, that acts *in trans*, and the inert, intronic part.

The pattern of diversity of group II introns is compatible with this scenario. Group II introns are found in ~25% of the sequenced bacterial genomes, a few archaeal genomes, and organellar genomes of fungi, plants, and protists [54,55]. However, the structural features and behavior of prokaryotic and organellar group II introns show telling differences. In prokaryotes, most of the group II introns contain intact open reading frames (ORFs), with the reverse transcriptase (RT), maturase, and in many cases, endonuclease domains, and behave much more like mobile retroelements than like introns. With a few notable exceptions, they do not insert into biologically important, conserved genes and, most often, actually insert into intergenic regions, hence, formally, losing the intron status. Prokaryotic groups II introns show signs of both intragenomic and horizontal mobility, with the homing mechanism involving reverse splicing and reverse transcription reactions.

The organellar group II introns are notably different. Most of them reside in evolutionarily conserved, essential genes and show various degrees of degeneration of the intronic ORFs[55]. The RT and endonuclease domains, which are involved in mobility, are typically disrupted whereas the maturase domain survives in a greater fraction of the introns. These are, indeed, typical introns, inasmuch as their splicing is required for the expression of the respective genes. In some of the organellar introns, the ORF is completely disrupted and splicing is facilitated *in trans *by a maturase encoded in a different intron. The mobility of these introns is limited although some might still occur within the same organellar genome. Thus, the organellar group II introns look conspicuously like functional and, perhaps, evolutionary intermediates between prokaryotic retroelements (as one should more properly call group II introns found in bacteria and archaea) and the eukaryotic spliceosomal introns. The major difference in the spread and properties of the bacterial and organellar group II introns is likely caused by the differences in effective population size which is several orders of magnitude greater for bacteria than it is for organelles [57].

So what happened at the onset of eukaryotic evolution? The scenarios of eukaryogenesis favored by different researchers differ dramatically, and this is not the place to review in any detail the pros and cons of each of these scenarios [58]. In any case, there is no doubt that the symbiosis between an α-proteobacterium, the ancestor of the mitochondria, and a somewhat mysterious host, the ancestor of the eukaryotic cytosol and nucleus, was a crucial early event in eukaryogenesis. A rather radical but, in my opinion, also the most parsimonious and, all things considered, the most realistic picture of eukaryogenesis is a non-mysterious one, namely, that the host invaded by the α-proteobacterium was a garden-variety archaeon (the case for this scenario has been, in part, presented recently [59] and will be discussed in full elsewhere). The α-proteobacterial endosymbiont must have brought with it a certain number of group II retroelements, and these, apparently, have gone berserk within the host cell [59]. The reasons for their sudden onslaught on the host genome are not entirely clear. Perhaps, one of the most important factors was, simply, the small effective population size of the "prekaryotic" chimera which precluded rapid elimination of the inserted retroelements by purifying selection that holds them at bay in large bacterial populations[60,61]. It is conceivable, also, that the "naïve" archaeal host lacked (still poorly understood) control mechanisms operating in bacteria to limit the spread of group II retroelements.

The spliceosomal introns that have changed to the extent that their ancestry is not immediately obvious and, in particular, have lost all the activities of prokaryotic group II introns/retroelements (i.e., the ability to catalyze their own splicing and reverse splicing) comprise one major line of descent spawned by the invaders in eukaryotic genomes. The other line consists of all the numerous eukaryotic retroelements that insert between genes or within introns [62,63]. Some copies of these retroelements retain the RT which provides for their mobility and survival.

### Multiple, pivotal roles of ancient introns in eukaryogenesis and redemption of the exon theory

The chimeric "prokaryote" could survive the attack of bacterial retroelements only if it evolved, in the very least: i) a mechanism of compartmentalization that separated the intron-containing transcripts from translating ribosomes allowing the relatively slow splicing reactions to occur before translation begins and thus precluding the formation of aberrant polypeptides, and ii) a reasonably efficient mechanism of splicing that acted *in trans *and hence was capable of efficiently removing even those introns in which the ORF was completely disrupted [59]. It is most tempting to infer that these requirements were the principal driving forces behind the evolution of two of the principal eukaryotic innovations, the nucleus and the spliceosome. Indeed, as recently detailed elsewhere [59], the original function of the nuclear compartment might have been isolation of pre-mRNA transcription and splicing from the cytosolic translation system such that unspliced transcripts would not be translated to yield deleterious, aberrant polypeptides. The advent of the spliceosome is even more straightforward, with the terminal segments of Group II introns recruited as snRNAs, the ribozyme part of the spliceosome, and the Sm protein, which is involved in RNA processing in archaea[64,65], becoming the spliceosome's protein core.

**Figure 2:**
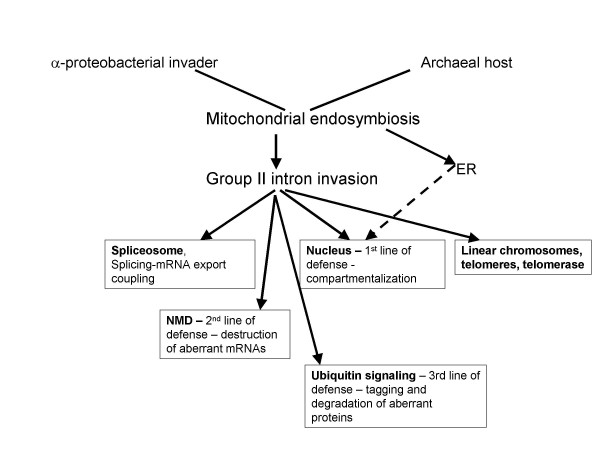
**The proposed chain of causes and events in eukaryogenesis – the pivotal roles of mitochondrial endosymbiosis and intron invasion**. Arrows indicate proposed causal relationships (selective forces).

The origin of the nucleus and the spliceosome might not have been the only innovations brought about by the retroelement onslaught. I propose here that several other major eukaryotic novelties were triggered by the very same "intron catastrophe" (Fig. 2). In particular, and in addition to the nucleus, the intron invasion conceivably precipitated the emergence of two additional lines of defense against the accumulation of abnormal mRNAs and protein. The second, after the nuclear compartmentalization, defense system is Nonsense-Mediated Decay (NMD) which performs "quality control" at the level of transcripts. The NMD encompasses a suite of nucleases and helicases that is conserved in all eukaryotes and eliminates aberrant mRNAs containing premature termination codons [66-68]. In particular, the NMD system has been shown to specifically destroy aberrantly spliced mRNAs [68-71]. The core of the NMD is thought to have evolved from a bacterial post-segregational cell-killing, toxin-antitoxin system which contains nuclease domains homologous to those employed in NMD [72]. Thus, it appears likely that a toxin-antitoxin system from the mitochondrial endosymbiont was recruited for the mRNA surveillance function at an early stage of eukaryogenesis. This would be particularly important because, at that stage, the coupling between transcription, splicing, and nucleocytoplasmic transport would not yet have been perfected, and leakage of (partially) unspliced transcripts into the cytosol would have been a substantial problem.

The third and last line of defense that is invoked if an aberrant transcript escapes surveillance and is translated appears to be the incredibly elaborate system of ubiquitin-based signaling which is one of the functional signatures of eukaryotes and is responsible for most of the regulated proteolysis and much of protein topogenesis in eukaryotic cells [73-76]. Although prokaryotes do their fair share of regulated protein degradation[77,78], they do not seem to have a direct functional analog or precursor to the ubiquitin system. The prokaryotic homologs – and apparent evolutionary progenitors – of proteins that, in eukaryotes, comprise the protein machinery of ubiquitin signaling, are components of the pathways for biosynthesis of molybdopterin and thyamin [79-82]. I propose that ubiquitin signaling originally evolved as a second line of defense against the intron invasion that, at least at the early stages of eukaryogenesis, would have led to an increase in the formation of abnormal proteins from translation of leaked unspliced pre-mRNAs, despite the emergence of the nucleus as the primary protective device. Hence the driving force for the evolution of a specialized mechanism to target these aberrant proteins for degradation. Protein "quality control" might have been the original role of the ubiquitin system and remains one of its crucial functions to this day [83-85], even as it evolved to assume many other roles.

Compared to prokaryotes, eukaryotes have a greater number of multidomain proteins that substantially contribute to the functional complexity of the eukaryotic cell [86-88]. It seems likely that the early, mobile introns could have contributed to the emergence of multidomain protein structure via recombination between (nearly) identical introns in different genes. Obviously, most of such events would be strongly deleterious but some might have created potentially useful domain combinations without losing much important information, and thus would be picked by selection. It remains to be investigated in detail whether or not ancient eukaryotic multidomain protein whose origin can be traced to the onset of eukaryogenesis tend to contain introns between the domains. Should it turn out that introns indeed contributed to the evolution of ancient, eukaryote-specific multidomain proteins, this would seem to be a new, more realistic incarnation of the exon theory of genes.

Another outcome of recombination between (nearly) identical introns located in distant regions of the chromosome could be disintegration of the circular chromosome of the archaeal host into multiple, linear chromosomes. Replication of linear chromosomes presents a problem as it leads to gradual loss of the terminal (telomeric) regions [89-91]. The solution is evolution of replenishable telomeres, and it is remarkable that the key enzyme of telomeric repeat propagation, the catalytic subunit of the telomerase, probably, evolved from the RT domain of group II introns[62,63,92]. Thus, intron invasion might have both caused the problem of linear chromosome replication and provided the solution.

In addition to all these innovations, the intron invasion, obviously, created the potential for controlled alternative splicing, a mechanism that came to prominence at a later stage of eukaryotic evolution and made a crucial contribution to the evolution of complexity in multicellular organisms. In a sense, the (potential) contributions of introns to eukaryogenesis that are outlined here recapitulate aspects of the exon theory of gene evolution. Indeed, although there seems to be no support for a role of introns in the emergence of the original genes, their roles in eukaryogenesis might have been multiple and crucial, in line with the gist of the exon hypothesis – the evolutionary importance of the "junky" intron sequences.

### Self-splicing introns as heritage of the primordial genetic pool

As discussed above, eukaryotic spliceosomal introns as well as core components of the spliceosome, most likely, have evolved from group II self-splicing introns that should be more appropriately characterized as prokaryotic mobile retroelements. There is every reason to believe that they are at least as old as prokaryotes themselves if not older. Of course, scenarios for the origins of the first cells are widely open to debate. Nevertheless, at least some of these scenarios – and, in my opinion, the more plausible ones – include some version of a primordial pool of mixing and matching genetic elements[93,94]. A particular model that has been elaborated recently seizes on the disparity between the membrane biogenesis and DNA replication systems in bacteria and archaea to propose that the primordial genetic pools, that might have thrived in networks of inorganic compartments at hydrothermal vents, were the form of life that prevailed on earth until the separate escapes of the archaeal and bacterial cells from these hatcheries[95]. This model involves selfish genetic elements competing for resources and, originally, selected solely for their ability to replicate efficiently, gradually evolving into "selfish cooperatives" that would include multiple elements coding for mutually beneficial functions (e.g., replication, translation, and nucleic acid precursor synthesis). Wherever there is cooperation, there will be parasites, and evolution of selfish cooperatives would inevitably spawn various types of parasitic genetic elements, the progenitors of modern selfish genetic elements and viruses[95,96].

Under this model, the primordial genetic pool is believed to have evolved from a pure RNA world to a RNA-protein system to the modern world of the Central Dogma (DNA-RNA-protein) through an intermediate stage, possibly, corresponding to the LUCA, at which a retrovirus-like cycle of replication became widespread[96,97]. At this stage, parasitic retroelements, the would-be group II introns, would inevitably emerge.

This line of reasoning leads straight to a simple but rather startling conclusion: introns that currently reside in eukaryotic genes, after all, do derive, ***through an uninterrupted lineage of selfish elements***, from primordial genetic elements. Hence at least a formal vindication of another aspect of introns-early: introns have evolved extremely early, very likely, earlier than cells themselves.

## Conclusion

**Figure 3:**
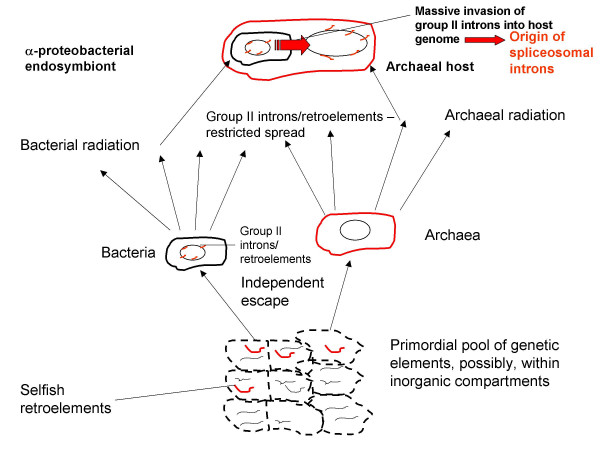
**A brief early history of spliceosomal introns**. The scheme shows the inferred sequence of events from the primordial pool of genetic elements to the origin of spliceosomal introns from group II introns invading the host genome upon mitochondrial endosymbiosis.

Through a synthesis of comparative-genomic data and bottom-up reconstructions of early stages of life's evolution, it is now possible to outline what I believe to be a fairly complete and credible history of spliceosomal introns (Fig. 3). According to this reconstruction, the evolutionary precursors of spliceosomal introns, the self-splicing group II introns, started out as parasitic retroelements within the primordial genetic pool and have been retained by the first bacterial (and, possibly, archaeal as well) cells. Throughout the entire history of the prokaryotic world prior to the advent of the eukaryotes, these retroelements maintained their relatively uneventful selfish existence, being kept in check by the strong purifying selection in large prokaryotic populations and, possibly, also by specific control mechanisms. The beginning of eukaryogenesis was marked by a massive invasion of group II introns escaping from the mitochondrial endosymbiont into the host, archaeal chromosome that formed the basis of the emerging eukaryotic genome. This intron invasion triggered the formation of the signature features of the eukaryotic cell including the spliceosome, the nucleus, the linear chromosomes with telomeres, and the systems of nonsense-mediated decay and ubiquitin signaling, either as devices for direct defense against introns or as inevitable consequences of the invasion (Fig. 2). A substantial fraction of the introns retained their ancestral positions in multiple, diverse eukaryotic species although introns in some of the ancestral positions have been lost whereas some new ones have been acquired. However, the temporal characteristics of intron loss and gain appear to be very different: whereas losses seem to occur in a, more or less, clock-like regime, gains, apparently, have been episodic and, possibly, be associated with transitional evolutionary epochs. This new understanding of the history of introns has a peculiar bearing on the nearly 30-year old introns-early vs introns-late controversy. The central idea of introns-early (exon theory) on the role of introns in the origin of first proteins does not seem to receive any empirical support. Of course, recombination between RNA molecules could have been one of the processes that contributed to the emergence of the genes for first proteins from smaller, peptide-encoding segments (e.g., [98]). However, there is no indication that putative primordial non-coding sequences that might have been involved in such recombination ever gave rise to introns.

On a more modest scale, though, the introns-early view appears to hold true, at least formally: precursors of introns, most likely, were inherited by modern life forms from the primordial genetic pool. This is, however, only a hollow vindication of introns-early. More importantly, it appears that introns did play a major role in the evolution of biological complexity albeit, apparently, not at the earliest stage of life's evolution, but rather at the outset of eukaryogenesis. Thus, there seems to be no real losers in the introns-early vs. introns-late debate as both views capture important aspects of the evolutionary reality.

## Reviewers' comments

### Reviewer's report 1

W. Ford Doolittle, Department of Biochemistry and Molecular Biology, Dalhousie University, Halifax, Nova Scotia, Canada

Eugene Koonin's paper, like many speculative reviews in evolutionary biology, presents two intertwined stories: an *evolutionary narrative *about past events and forces that produced a particular aspect of the biological world, and a *history of science narrative *about how we as a community have come to hold our present beliefs about such events and forces – beliefs Koonin now hopes to reformulate. Koonin's evolutionary story, at least up to the part where introns drive the evolution of the nuclear membrane, is timely and, in my view, largely sensible. Most workers in the field (Gilbert and his colleagues excepted) now endorse the general view about introns Koonin articulates here – that all spliceosomal introns descend from Group II introns *introduced into *the nuclear genome of the last common ancestor of all extant known eukaryotes, by the prokaryotic ancestor of the mitochondrion.

*Author response: Before addressing some of the specifics of this review, I must note up front that it is a great honor and a very special occasion to have this manuscript reviewed by one of the originators (along with Jim Darnell) of much of our understanding of introns and their evolution, and the author of the original terms. I try to interfere as little as possible with the historical part of this review, to let the reader enjoy the narrative. To me at least, this is truly exciting reading*.

I think that from the history of science perspective, Koonin has the early days of *introns early *about half right (an excellent score, by the way, given the inherent difficulty of the task). Since I actually coined the terms *introns early *and *introns late *(in a 1987 review not often cited [American Naturalist 130: 915–928]), I would like to claim some special knowledge as to how these theories first arose and have evolved, and some special right to judge what current data have to say about the truth of them. No theory is exactly what it was when first formulated. Theories evolve, and are scarcely if ever confirmed or refuted in their original forms. So part of the process of science is negotiation over whether the current form is the natural child of the original, and thus whether a theory's originators can ever legitimately be said to have been right or wrong. Such negotiations are not easy: it's [only] a wise father that knows his own child.

*Author response: Sadly, I was unaware of the American Naturalist review as well (not in PubMed). To keep the record straight, I am not citing it in the body of the article – here is the citation for the interested reader, in this review, from the author himself*.

So here's how *I *recall the theory's history. In his 1978 "*Why genes-in-pieces*" Wally Gilbert famously argued that the mosaic structure of eukaryotic genes allowed them to evolve by a subtler mode and faster tempo than could the genes of prokaryotes, through what came to be called *exon shuffling*. This was not yet his *exon theory of genes*, which was named by him in 1987 and incorporated much of his and others' subsequent thinking about the "RNA World" into a general view about gene origins. Indeed, "*Why genes-in-pieces*" did not address origins, or seek to explain the presence of spliceosomal introns in (all) eukaryotic nuclear genomes and their absence from (any) prokaryotic genomes. The prevailing notion at the time, however, was that eukaryotes emerged *from within prokaryotes*, through the coming together of several lineages (the Serial Endosymbiosis Hypothesis of Margulis and Taylor) and that the eukaryotic nuclear genome is a complexified descendant of a prokaryotic one. Parsimony would thus have dictated that introns were introduced into once-intact prokaryotic genes at the time of this prokaryote-eukaryote transition.

That is to say, had "*Why genes-in-pieces*" addressed intron origins within the then reigning phylogenetic paradigm, it would have been most akin to *introns late*. But it didn't, and origin issues arose first in attempts to explain how and why (by what mechanism and in response to what selective forces) mosaic gene organization first came into being. After all, exon shuffling is only useful after many introns have been introduced. And even then the benefit is a long term one: evolution does not look ahead and the short-term cost would have been prohibitive. So their presence was an evolutionary mystery.

A way around this was to imagine that eukaryotes had not emerged from within prokaryotes after all, and were instead a parallel line of descent anciently diverged from them at some inchoate stage of cellular and genomic evolution. Then we might with equal respect for parsimony see introns as a primitive genomic feature, since lost from prokaryotes (through "streamlining"), but retained in eukaryotes, where they could indeed confer the evolutionary advantages mapped out in "*Why genes-in-pieces*". A few of us had already been thinking along just those lines even before the discovery of introns. Jim Darnell, in particular, had already speculated that the bizarre and seemingly very wasteful things that eukaryotes do with mRNA bespeak retained primitivity. To him (and to me) the elegant efficiency of prokaryotic machinery for gene expression seemed the more evolutionarily refined, with the loss of introns being but part of this refinement. For me a crucial additional component in making this "eukaryotes early" notion seem reasonable was the articulation of the three domain concept by George Fox and Carl Woese, and their redrawing of the Tree of Life (which happened pretty much simultaneously with the discovery of introns in eukaryotic nuclear genes, in 1977). In the first versions of this tree and until the late 1980s, the three domains emerge independently from a primitive ancestral state ("the progenote").

So the absence of introns in prokaryotes was not as Koonin asserts "a potentially embarrassing complication" needing to be "explained away" but rather an important element in the development of the theory! In the context of the unrooted three-domain tree, *introns early *was no more "a decidedly unparsimonious scenario" than was *introns late*. Indeed, if introns played the role in gene assembly first envisioned or later integrated into the RNA World hypothesis by Darnell and me (PNAS 83: 1271–1275) and by Gilbert in his "*Exon Theory of Genes*", their presence in the progenote and before may even have been a precondition for the rapid evolution of complex proteins. Really we know nothing about how genes arose, and to suppose that they sprang full blown and full length from noncoding polynucleotides seems to me more of a stretch than to imagine that they were cobbled together from smaller oligopeptide-encoding modules. Parsimony is often in the eye of the beholder, and its relevance in reconstructing evolution is in any case questionable. Surely we do not require of real historians that a primary criterion for constructing narratives about the human cultural or political past be that they invoke only the minimum possible number of historical forces and events! Yet what are we biological evolutionists but historians of Life?

*Author response: To get the history at least closer to the real picture, the introductory text was slightly modified to emphasize that the full-blown exon theory is a later development than the original introns-early. With regard to the more substantial aspect of this comment, I must agree that we know (next to) nothing about the emergence of proteins. Assembly from short peptides is one of the possible routes and (autocatalytic) recombination between RNA molecules very well could have contributed to that. However, this is not the same as introns-early. Inasmuch as there is no evidence of an evolutionary relationship between such putative non-coding sequences and modern introns, this seems to be a different scenario. Origin of proteins is, generally, outside the scope of this paper (it is relevant only inasmuch as introns are implicated) but a clarifying comment on this issue was added to the Conclusions*.

*On a more general note, the status of parsimony in reconstructions of the past and the comparison to the historians of the human civilization ("real historians") seems to beg for some comment. I believe the role of parsimony depends on the depth of history one considers. Surely, it would be a little preposterous to claim that, in February of 1917, Lenin's Bolshevik party snatched the power in Russia directly from Nicholas II, on the grounds that considering any intermediate between the two systems would violate parsimony. That is because we know for a fact that, in February of 1917, the power was transferred to the pluralist Interim Government, and it was only in October of that year that the Bolsheviks took over, through an improbable combination of events. And yet, what guiding principle do "real historians" have when it comes to early stages of the history of civilization, where the only evidence available is archaeological (along, perhaps, with some comparative linguistics)? I believe one has no choice other than to rely on parsimony or, put another way, Occam razor. The same applies to the reconstructions of biological evolution, so I do not really agree that the role of parsimony is questionable. It is, indeed, another and, I think, more difficult matter that parsimony is "in the eye of the beholder", i. e., we often effectively apply weighted parsimony, with the weight of different characters determined either on pure intuition or with some rationale. Herein seem to lie many problems in deep reconstructions, and much caution is needed*.

What tipped the balance against *introns early *– for me anyway – *was *nevertheless a powerful parsimony argument, based on a revision of the phylogenetic consensus. By the late 1980s, we had come generally to believe in a *rooted *three-domain tree, with archaea and eukaryotes sisters, and bacteria more deeply diverging. So now we *introns early *advocates would have to imagine two independent episodes of intron loss through streamlining (one in the line leading to Bacteria and the other on the line to Archaea) rather than the arguably more parsimonious single gain early in eukaryote evolution imagined for *introns late*. More importantly, by then we'd come to believe that the (seemingly) most deeply diverging eukaryote lineages (*Giardia*, *Trichomonas *and the microsporidia) lacked both mitochondria and introns. Independent loss episodes would have to be imagined for each of these several eukaryotic lineages in order to hold on to introns early. John Logsdon and Jeff Palmer were the first to make us (or me anyway) aware of the serious unparsimoniuosness of this scenario. And if, as then seemed likely, eukaryotes with mitochondria had introns and those which had never acquired such organelles didn't, why not suppose that the latter were introduced along with the former, as Tom Cavalier-Smith did, in 1991. Introns late acquired a vector for the introduction of introns, and a specific scenario for their origins as genetic entities, from Group II introns in bacteria.

But, since the turn of the millennium, the balance of phylogenetic arguments for or against *introns early *has shifted again, returning to the neutral position! All eukaryotes likely have introns and likely all have, or once had, mitochondria. So Koonin argues that the door is open again for *something *like *introns early*. However, it does matter what we take that term to mean. Rather early on in the *introns early – introns late *debate, it became clear to most protagonists that the notion that *all *introns are primoridial relics, with a history exclusively of loss, would not wash. Lining up all the positions at which introns are known in at least one of all the available orthologs of a given gene forces the conclusion that ancestral exons were far shorter than needed to code for any credible protein structural module. For me, the "weaker" or "revised" version of *introns early*, the one under dispute for most of the two decades before the turn of the millennium, and in my judgement the only legitimate child of the 1978 version of the theory, was this: the first genes were assembled from smaller protein-coding modules, introns marked the positions at which such modules were joined, and at least some introns (or intron positions) in some modern eukaryotic nuclear genes are direct relics of that assembly process. So even if LECA's genes were chockablock full of Group II introns or their degenerate spliceosome-dependent products, as long as we see these introns as having been "introduced into" the nuclear genome (producing the explosive infectious spread Koonin calls the "intron catastrophe"), we're dealing with an *introns late *scenario.

But do we absolutely have to see it that way? We believe that eukaryotes and archaea are sisters because we believe that the genes that produce this sisterhood relationship, which are mostly those of translation and other components of the information processing machinery, tell the true story of eukaryote origins. And we see the non-archaeal genes in eukaryotic genomes as having been "introduced into" the eukaryotic cellular lineage because we still see the pre-mitochondrial (bacterial) cellular lineage, from which many of these genes likely derive, as having been introduced (as an "endosymbiont") into some post-archaeal host cellular lineage. Or most of us see it that way, anyway.

But eukaryotic origin scenarios in which the complexity of eukaryotic cells and their ability to engulf other cells arise only after the tight integration of the bacterial and archael parental lineages are increasingly popular (see Embley and Martin, 2006, Nature 440: 623–630). In these scenarios, designation of one lineage as host and the other as symbiont (and the notion that one set of genes was "introduced into" the other), seems pretty arbitrary. Indeed, if we chose to go with the majority of genes and accept the analyses of Esser *at al*. (2004, Mol. Biol. Evol. 21: 1643–1660) that suggest that the majority of yeast nuclear genes are of bacterial ancestry, then surely we must seriously entertain their ironic conclusion that "yeast shares a sister-group relationship with eubacteria, not with archaebacteria".

The bacterial lineage and many its genes presumably came already modestly freighted with introns, albeit Group II introns. There is no reason to assume that all spliceosomal introns that now dot the eukaryotic nuclear genomic landscape landed there as the result of *de novo *events of transposition of such Group II introns. Put another way, there is no reason not to assume that some of the Group II introns in genes that are part of the bacterial heritage were simply converted, *in situ*, without moving so much as a nucleotide to left or right, into the pitiful degenerates we now call spliceosomal introns. Such a scenario is, *mirabile dictu*, perilously close to *introns early *in what Koonin calls "its modified form" but considers untenable, favoring "a massive invasion of Group II introns escaping from the mitochondrial endosymbiont into the host".

But this scenario is indeed not the same *as introns early*, unless we in addition suppose that some individual bacterial Group II introns that have survived as spliceosomal derivatives in eukaryotic nuclear genes were themselves relics of primitive stages of gene assembly. This may seem unlikely, given that in bacteria Group II introns appear to avoid the interior regions of protein coding genes. But who knows? Maybe bacterial genomes were once riddled with relict introns, some indeed primordial leftovers. Maybe these were removed one at a time as translation got increasingly fast and waiting for splicing increasingly a burden, and remain mostly where interference with translation is not a big issue. The imposition of the nuclear membrane, far from being a defense against invading introns, might have allowed the relatively few such parasites that were part of the bacterial heritage to run amuck. So it would not be that the nuclear compartmentalization arose as a barrier to the "attack of bacterial retroelements" but rather that it arose for some other reason, and *permitted *the subsequent proliferation of introns.

None of us can present his or her evolutionary hypotheses without embedding them in a story of the relevant thinking that has gone before, else we would be plagiarists (or so off-the-wall that our ideas bear no debt whatever to the past). But each of us has a different take on that past, and each of us recounts it so as to make his/her own contribution seem to be the product of inexorable logic – not just for rhetorical purpose but because this is how we come to understand it, ourselves. Koonin's arguments about the biology are interwoven with his recounting of the history of thought in the area, in service of his goal to determine whether a 38 year old theory is still worthy of consideration because it most parsimoniously explains the present data. Whether or not the *introns early *theory is judged to be parsimonious (and thus more or less believable) depends on what one takes to be the relative likelihoods of the inferred genetic processes (streamlining *versus *massive intron infestation) and the accepted phylogenetic framework (the Tree of Life) against which its parsimoniousness should be evaluated. The former is unknowable and the latter has varied radically over the life of the theory, and still varies radically between individual theorists in the field.

Koonin's evolutionary narrative, as I understand it, is this:

• Genes were probably first assembled from pieces, as envisioned in the early versions of *introns early *and later elaborated in the "exon theory of genes".

• Group II introns, as a class of transposable selfish elements, may well date from this time of gene assembly. They continue to infest bacterial genomes, albeit at modest level, and the (endosymbiotic) bacterial genome that became the mitochondrial genome was so infested.

• The host ("archaeal") genomic lineage was (for some reason) devoid of such elements, which were introduced into it by transposition from this (endosymbiotic) bacterial genome. The defenseless host genome became the scene of a transpositional orgy.

• Compartmentalization of the nuclear genome and several other aspects of eukaryotic cell biology arose as defenses against this "intron catastrophe".

*Author response: This is an important point, and clarification is due. The first statement among these four is not an integral part of my narrative. I think that the issue of the origin of first proteins is wide open (see above) and recombination between RNA molecules is one of the likely contributed mechanisms (again, see the revised Conclusion). However, this is not where I see the link to – and partial vindication of – introns-early. This connection, mostly, stems from points (ii) – the evolutionary antiquity of the (precursors of) introns and (iV) – the crucial role of introns in eukaryogenesis*.

He claims that this scenario "combines aspects of *introns early *and *introns late *views" which it does, and that there may be "no losers in the *introns early vs. introns late *debate as both views capture important aspects of the evolutionary reality." I'm not sure, however, that "aspects" are enough to legitimize Koonin's version of *introns early *as the natural child of the 1978 theory. In my opinion that theory, even in its weak form, requires that "at least some introns (or intron positions) in some modern eukaryotic nuclear genes are direct relics" of primordial gene assembly process. (By 'direct relic' I mean occupying the original position in the genes and descendant through replication.) That so many introns have been added since, from whatever source and by whatever mechanism, may mean that the signal is too small to be detected and the theory thus formulated is unfalsifiable. This doesn't mean that it is false, or even useless, although it may no longer be a *scientific *theory. There are many interesting ideas out there about how life itself first arose. Few of them (only the chemically impossible) are falsifiable, but one or more of them may have elements of truth, and we do know that life did, somehow, arise. And thinking about such theories stimulates the collection of much data and the generation of many new ideas, as have *introns early *and *introns late*, in their 38-year struggle for the hearts and minds of molecular evolutionists.

*Author response: Parts of the manuscript, including the title (as per Bill Martin's suggestion), have been modified to de-emphasize the claim that the debate ended in a "draw". I really do not insist that the current view of the evolutionary history of introns articulated in the present paper is a true, legitimate child of the original introns-early. I only would like to maintain that there is a connection, i.e., some heritage of that view factors in the currently emerging picture. If the latter is a true child of introns-late, but just a nephew of introns-early, that's fine with me*.

### Reviewer's report 2

James E. Darnell, Laboratory of Molecular Cell Biology, The Rockefeller University, New York 10021, USA (nominated by W. Ford Doolittle)

I think speculation on the origin of eukaryotic cells that does not go back to the pre-cellular phase of evolution unlikely to unlock any secrets and at worst to be vacuous. Therefore I warm to Koonin's last section, "Self-splicing introns as heritage of the primordial gene pool." To quote from Carl Woese, "Next comes the evolution of the eukaryotic cell itself. While biologists have traditionally seen this as a step (saltation) beyond the stage of bacterial cells, I do not." ["A new biology for a new century", Microbiol. Mol. Biol. Rev. vol. 68, 173 (2004).] He then goes on to develop the vast difference in cell structure and organization (and inferentially structural proteins). His objection to eukaryotic cell development AFTER non-nucleated cell development might be thought to dissolve with the discovery of proteins in bacteria that have the same three-dimensional structure as tubulin and actin [Lowe et al. (2004) Molecules of the Bacterial Cytoskeleton. Annu. Rev. Biophys. Biomolec. Struct. 33, 177; Amos et al. (2004) Structural/functional homology between the bacterial and eukaryotic cytoskeletons. Curr. Opin. Cell Biol. 16, 24]. However that does not undercut the more general argument for pushing eukaryotic origins back to a very early stage of cell evolution. What all this suggests to me is that essentially all of the protein folds existed by the time sustainable 'life' as we know it arose. If this is the case, then the platform from which eukaryotes arose could be the same as that from which the other two kingdoms arose. And then the question is what the pools looked like from which the extant genomes were constructed. What strikes me now (and always has) is the unlikelihood of already highly developed, single, non-nucleated cells whose aim in life is to grow and divide being the entities from which eukaryotes derived. It seems much more plausible to me to back up and admit that pre-cellular 'life' had passed the Rubicon of coded protein (peptide) synthesis and probably elementary nucleic acid duplication (even DNA synthesis). In this gemisch the quite different polymer synthesis machines, archaeal and bacterial, must also BOTH have existed before functioning cells. And if this pre-cellular stage provided the platform from which the two (and only two?) non-nucleated cell types arose, multiplied and remained distinct (ignoring LGT), it is from this same pre-cellular but elaborate stage that I ^3^believe^2 ^(too strong and irrelevant a word) that eukaryotes arose carrying with them somehow the ability to get along with fragmented nucleic acid (or at the very least self-splicing intron introduction and removal capacity), an ability with which the whole pre-cellular pool must have been endowed and which has been maintained in all multi-cellular eukaryotes and by inference the one (or more?) single-celled precursors to multi-cellular eukaryotes. Surely small bits of information predated large chunks of information and somehow tacking together sufficiently large pieces of information to make usable peptides was an early (? primary) task (See for example the idea of an amoeba-like ancestral protozoan; maybe we should use progenote, the two decade old word.) that 'could serve as a genetic melting pot.' (Ogata et al. 2006. PLoS Genetics 2, e76, May 12). Lacking more insight into this stage leaves me unwilling to swallow a primitive self-sustaining eukaryote devoid of a mechanism for recruiting useful information from disjointed stretches that then swallowed and used non-nucleated cells as plastids and gene donors. The latter swallowing surely occurred but by whom? I realize that this position is not particularly helpful since it generates few (? no) experiments/measurements/observations, but to discuss eukaryotic origins as obligatorily arising from either bacteria or archaea or both seems to me wrong-headed. Eugene Koonin's paper is reasonable, written well and highly informative on many up-to-date details. But its diagrammatic simplicity embracing the 'later' dependent origin of eukaryotes doesn't convince me that a reasonable proposal is a right proposal.

Author response: I appreciate the thoughtful comments from one of the founding fathers of our current understanding of eukaryogenesis. I think there are, actually, many points of agreement with respect to the evolution of many complex features of life, including the two distinct DNA replication machineries (archaeal and bacterial) within a precellular genetic pool (see our paper with Bill Martin on this subject, ref. 95). What we seem to disagree on is the early (from within the same pool) vs late (via archaeal-bacterial fusion) origin of (pro)eukaryotes. For sure, a reasonable proposal is not necessarily a right proposal. But should we not try to stick to a reasonable proposal and explore its implications until it is shown to be false?

### Reviewer 2's response to replies

James E. Darnell, Laboratory of Molecular Cell Biology, The Rockefeller University, New York 10021, USA. (nominated by W. Ford Doolittle)

You have reason on your side.

### Reviewer's report 3

William Martin, Institute of Botany III, University of Dusseldorf, D-40225 Dusseldorf, Germany

This is an interesting and informative paper, although I don't think that the current title is very good. Introns early and introns late are irreconciliable views, they can't both be right so it is not really a draw. And if they are both wrong it is not a draw either. The paper is basically an essay on "some aspects of eukaryotic introns and their possible evolutionary significance" and a title along those lines might better advertise its content. That title would paraphrase one of Ford Doolittle's more famous titles (54th Symp. Soc. Gen. Microbiol. Pp. 1–21, 1996), which was intentionally identical to Stanier's famous 1970 SGM title (Symp. Soc. Gen. Microbiol. *20*, 1–38). Too bad that there is not a 2006 SGM symposium volume, this would have fit nicely.

*Author response: This point is appreciated and well taken. So much so that the title of the paper has been modified along the lines suggested. The new title actually seems to give better justice to some of the more substantial (as opposed to historical) aspects of the present paper. For the reader's understanding: the original title was 'Introns-early versus introns-late: is it a draw?' I agree that this is not a "real" draw, and if we formulate the debate as an 'either/or' question, we have to accept that introns-late has won. However, the version of introns-late that is currently taking shape has very strong reverberations with introns-early, hence the original title. The same thought is expressed in the revised title in a more cautious manner. Several changes to the wording in the Abstract also have been made, to go along with the new title. Having changed the title, we could decide to dispense with all this discussion but I think it might be of certain interest to the reader and worth keeping in the published record*.

As its main thrust (from my viewpoint), the text extends and further develops the idea in ref. 59 that introns may have precipitated the origin of the eukaryotic nucleus (as a consequence of the origin of mitochondria) and NMD by the compatible notion that introns might have precipitated ubiquitinylation as well. This suggests that several otherwise puzzling eukaryotic novelties can be *better understood *(under these premises) as defense responses in the broad sense against invading mobile elements (group II introns, from the mitochondrion in the simplest interpretation). Ideas that aid our *understanding *need not require that the ideas are correct (how can we prove anything about early evolution anyway?). But if they help us to structure the problem (and the prokaryote-to-eukaryote transition needs considerable restructuring with the demise of archezoa) by linking phenomena that appear otherwise unrelated in a temporal and mechanistic manner that is tangible, then that constitutes progress. This paper fills that bill.

*Author response: I appreciate this comment and, by and large, agree. This is what I was trying to do, basically, to advance, even if just a little bit, our understanding of the wondrous series of events associated with eukaryogenesis. A full-scale philosophical discussion certainly is out of place here but it is tempting to throw in a brief comment. I agree that it is impossible to "prove" anything about early evolution but, then, this applies to most general statements about the physical world, even those that have nothing to do with remote past. Typically, such statements are accepted as "correct" inasmuch as they i) are compatible with a large body of diverse empirical data and ii) explain diverse aspects of the world in an economical and plausible manner (of course, this is a rehash of the well-known Popperian paradigm, I have no aspiration to be original here). With regard to early evolution, it is, most of the time, particularly hard to properly satisfy criterion (i) although we certainly must keep trying. Accordingly, criterion (ii) is becoming particularly important*.

At the same time, the paper looks further back in evolution towards the origin of genes and aims to reconcile aspects of introns-early with aspects of introns-late. The important evidence for intron stasis in animals indicates that one aspect of introns late in some of its original formulation is wrong: introns are apparently not continuously mobile over time. There was a time when they were actively mobile, but that time has apparently past. I still find it interesting that, as far as I know, nobody has ever observed the same intron in two places in genomes, that is, recent spliceosomal intron transposition does not seem to occur at an observable rate. Introns late was saying that it should be ongoing today. One point for introns late, but probably for the wrong reason. One could probably calculate the maximum rate at which introns with significant sequence similarity move, that would be interesting.

*Author response: Generally, true: unlike intron loss that is, more or less, clock-like, intron gain does not at all seem to occur uniformly in time, which is bad news for a strong version of introns-late. This being said, there are indications of recent intron gain in some lineages, and the reference to Coghlan and Wolfe (Ref. 53) on intron gain in nematodes was added in support of this point*.

If one accepts the view of an RNA world, then recombination is splicing, so the idea that splicing was around very early fits well. The idea that spliceosomal introns are in situ holdovers from that phase of gene invention (the exon theory of genes) is part of the eukaryotes early (or thermoreduction) idea still today, but beyond the New Zealand and French sections, I don't know that very many folks are card-carrying members in that club anymore. Ref. 98 distinguishes introns first from the exon theory, that could be debated because except for snoRNAs they are saying the same thing. Ref 98 has "intron gain" in the title, suggesting that there is some evidence for recent intron gain, I wonder what that evidence is, in detail.

*Author response: I tend to agree that the difference between 'introns first' and the good, old 'introns early' is minor, so to avoid confusion, I do not really discuss 'introns first' as a separate concept. As for evidence of gain, even apart from the Coghlan-Wolfe observations on the recent gains in nematodes, I believe reconstructions provide enough evidence in support of intron gain, at least at some stages of eukaryotic evolution. Granted, this is not the same as a "smoking gun", which would be a pair of highly similar introns in unrelated genes indicating a recent gain (like the Coghlan-Wolfe results, only more definitive). However, if intron gain was, indeed, episodic in eukaryotic in eukaryotes as suggested in this paper and previously elsewhere (ref. 50), such unequivocal cases of gain are likely to be very few and far between, and this seems to be so. Complete analysis of this issue, obviously, is beyond the scope of the present paper. Very hopefully, elsewhere*.

The history aspects of the introns early vs late debate are more easily dealt with accurately by someone who was in the thick of it. I'm not convinced that the whole introns debate is needed to understand the novel aspects of this paper, getting to the point more quickly might keep readers on track more effectively. Introns early vs introns late is over and I would not recommend warming back up. The present title as a subtitle of a more informative title might get the early introns players back into the arena again, though, which would be interesting. But this paper is suggesting that they would need to be debating other ideas, which is unlikely to occur.

*Author response: Maybe it would be easier and faster to cut to the chase right away and only discuss the current ideas on the role of introns in eukaryotic evolution without reheating the old debate...but I thought revisiting it was interesting. Ford Doolittle's review comes from the very thick of it and corrects my historical aberrations where necessary*.

Background, 2^nd ^paragraph: the exon theory of genes came much later

*Author response: Technically, yes, but the main ideas were there, I think. In any case, modified to reflect history more accurately*.

Results and Discussion, section "Multiple, pivotal roles of ancient introns in eukaryogenesis...". 4^th ^paragraph: exon theory of genes, but one that operates in eukaryotes only.

*Author response: Yes, this is the gist of it, more or less, as noted in the text*.

### Reviewer 3's response to the replies

William Martin, Institute of Botany III, University of Dusseldorf, D-40225 Dusseldorf, Germany

The replies to all comments, in particular the comments of referees 1, 2, and 4, add quite a lot to this interesting paper that was missing in the first version. This document now contains the best and most accurate summary of whence introns early that there is, but as a published referee report, that's something new for the literature. I wonder how to cite it.

What is the consensus of what has been said here? I think it goes like this: Because RNA recombination was probably involved in the origin of genes, and because splicing *is *RNA recombination, introns were probably involved in the primordial assembly of genes. But (NB: none of) the introns of eukaryotes were present at any of their current positions as those first genes arose, because spliceosomal introns are a secondary invention of the eukaryotic lineage via degeneration of group II introns that were acquired via the mitochondrion. This is not what either introns early or introns late was saying, as far as I can recall. But that debate structured the problem in a way that gave us concepts to sort the current observations from genomes into a reasonable temporal sequence of imaginable events. So I still think that the compromise offered by the title is not delivered. The present view is not introns early or the exon theory of genes, it is more the intron theory of nuclear origins. Any way one cuts the cake, introns still seem to carry burgeoning evolutionary significance; that comes closer to what introns early was saying than to what introns late was saying.

The view expressed here that the host for the origin of mitochondria was a garden variety archaebacterium (lacking introns, lacking a nucleus, lacking cytoskeleton, lacking phagocytosis) is a significant part of the present synthesis. This is what some of us have been saying for quite a while, the idea can be found in Ford Doolittle's 1996 SGM paper and in papers by Jim Lake (*Proc Natl Acad Sci USA *95:6239–6244, 1998; *Nature *431:152–155, 2004), Dennis Searcy (In *The Origin and Evolution of the Cell*. Hartman, H. and Matsuno, K. eds. World Scientific, Singapore. pp 47–78), and others (*Nature *392:37–41, 1998; *BioEssays *21:99–104, 1996). The significance of this concept is substantial because it implies that there *was no *lineage of eukaryotic-type gene or cell organization that goes all the way back to the origin of life (an assumption implicit in most formulations of introns early). The view of an archaebacterial host suggests that eukaryotes arose directly from fully-fledged and free-living prokaryotes, not directly from the same collection of organic molecules that gave rise to prokaryotes. It is thus mutually exclusive with regard to the progenote concept, which is closely related to introns early, but is compatible with some (but by no means all) symbiogenic views of eukaryogenesis. An historian of science specialized in the area of endosymbiosis, Jan Sapp, has gotten this aspect of the modern history of endosymbiosis and early cell evolution wrong on several occasions, so it was nice to see it spelled out more clearly by the authors and referees of this contribution. In 1981, Woese published an interesting paper in the June issue of *Scientific American *(p 98ff) that put his progenote view of eukaryote origins against Margulis's view of eukaryote origins head-to-head in figures. The progenote view was the view held by introns early, I suppose, or introns early might have even been an edifice of the progenote concept, who knows. Twenty five years later, neither the Woese camp nor the Margulis camp has indicated any willingness to find the kind compromise that Koonin has suggested here, as inspection of their more recent papers will attest. Maybe refugees from those two camps can someday find a popular solution – not a compromise! – that squares off well with the observations from genomes.

### Reviewer's report 4

Anthony M. Poole, Department of Molecular Biology and Functional Genomics, Stockholm University, Stockholm, Sweden

(a modified version of this review is to be published as a separate Commentary in Biology Direct)

This article comes in two parts; the first provides a thorough and up-to-the-minute review of the existing literature on the introns-early/late debate, is easy to follow and refreshingly even-handed for a field where one is too often subjected to vehemently partisan advocacy. This part of Koonin's article is well written and, while there's always something to disagree with in this field, I have no particular axe to grind. The second half of the article offers some new material, extending ideas Koonin developed with Martin (Martin & Koonin 2006 *Nature 440*:41).

*Author response: Again, before addressing some of the specifics, I want to emphasize that I greatly appreciate this careful, detailed, and constructive review. If my responses are (relatively) brief and I do not address all the points, just those I felt were the most important ones, the reason is my wish to avoid the specter of infinite regression that, indeed, plagues some philosophy journals*.

There is plenty in the second part of the paper to take issue with. In an attempt to avoid the phenomenon in philosophy journals where nit-picky rebuttals sometimes run longer than the original argument, I will confine myself to two main ones. The first is that, to buy any of this, one has to accept two main assumptions, both of which one can take issue with. If these do not hold, the entire argument comes crashing down. The second is that I do not think a good argument can be made that introns proliferated in the host that Koonin invokes in his model.

So, what are the assumptions we must accept? The first is that group II introns are related to the spliceosomal snRNAs and spliceosomal introns, and that the evolution of the these from group II introns is established, as per the mitochondrial seed hypothesis (Logsdon 1998 *Curr Opin Genet Dev 8*:637). The assumption of common ancestry is not really debated, since both introns-early and introns-late proponents accept this as given, albeit in different forms. In the former case, it has been argued that group II introns are relics of the RNA world (Gilbert & de Souza 1999 In: Gesteland et al *The RNA World*) and interrupted RNA genes, and that excision of the group II intron was required for production of functional RNAs. Group II introns would thus be early parasites, and, contrary to the historical situation Koonin alludes to where introns-early and the exon theory of genes were one and the same, this is not so in the revised introns-early scenario. However, in this scenario a satisfactory explanation for how group II introns could have evolved into a trans-splicing system comprising 5 snRNAs is not given, though, aside from an issue of timing, the problem is one and the same as for introns-late: the origin of the spliceosome.

Mechanistically, support for introns-late rests on the observation that group II introns-in-pieces are found (admittedly in a chloroplast genome – that of *Chlamydomonas reinhardtii*), and this three-piece self-splicing intron does at least provide a plausible intermediate in an organelle of bacterial endosymbiotic origin (see Stoltzfus 1999 *J Mol Evol 49*:169 for a model). The importance of this observation in the context of the mitochondrial seed hypothesis cannot be denied; other RNA genes in pieces are found in mitochondria (e.g. tmRNA – Keiler et al 2000 *PNAS 97*:7778), even if no split group II introns have thus far been found in this type of organelle.

The evidence for a common origin for snRNAs and group II introns is, by necessity, circumstantial; a common origin is invoked on the basis of the chemistry of reaction, which is in itself a weak argument (see Weiner 1993 *Cell 72*:161 for a critique). More significant is the demonstration that U5 snRNA can substitute for the ID3 domain of a group II intron (Hetzer et al 1997 *Nature 386*:417). Another piece of evidence comes from recent structural comparisons of the D5 domain of a group II intron and U6 snRNA (Sashital et al 2004 *Nat Struct Mol Biol 11*:1237; Seetharaman et al 2006 *RNA 12*:235), though finding similarities between two hairpins, while suggestive, is unfortunately not strong evidence for a common origin.

Overall, Koonin is being fair to the literature in so far as these similarities have all been used to argue for a group II intron origin for the spliceosome, and, as noted above, group II introns-in-pieces might well be expected to emerge in the mitochondrion. There is no question in my mind that, compared to models offered by the introns-early camp, the mitochondrial seed hypothesis is the better-developed hypothesis. It can potentially explain the stepwise emergence of a trans-splicing system comprised of several RNAs from a single cis-splicing element (though the large complement of proteins is more difficult – see Collins & Penny 2005 *Mol Biol Evol 22*:1053), whereas the exon theory of genes was never a good explanation for the origin of introns. It was quickly realised that this suffered from evolutionary foresight; introns would have had to have evolved in order to promote shuffling of domains, thereby producing new protein variants that would, possibly, be useful at some future point (Blake 1978 *Nature 273*:267; Doolittle 1978 *Nature 272*:581). That the exon theory failed to explain the origins of introns does not however mean that introns must by implication be late however (Gilbert & de Souza op. cit.; Poole et al 1999 *Bioessays 21*:880).

Indeed, it is unfair to argue, as Koonin does, that suggesting spliceosomal introns as a feature of the Last Universal Common Ancestor is indefensible because there are no introns in archaea and bacteria. As Koonin points out, group II introns are found in both bacteria and archaea, and, if, as widely supposed, spliceosomal introns & snRNAs and group II introns are descended from a common ancestor, the prediction that genome streamlining in archaea and bacteria eliminated spliceosomal introns can be reconciled with the presence of group II introns in these groups. Under streamlining, group II introns represent the intronic survivors of a period of reductive evolution. There is no direct evidence to support the contention that the ultimate ancestor was a group II intron, rather it is an assumption invoked as fact by many introns-late proponents, and repeated here by Koonin.

*Author response: If I understand the idea correctly, it is suggested that there was a stage of evolution of the hypothetical ancestral eukaryotes when Group II introns interrupted many genes. There is nothing inherently impossible about that. Moreover, there is a clear parallel with our (with Bill Martin) scenario under which such a stage was a transient one triggered by endosymbiosis. I believe, though, that the latter version has distinct advantages in terms of compatibility with the available data and explanatory power. Firstly, organisms with numerous Group II introns in protein-coding genes (to be concrete, with a density comparable to the density of spliceosomal introns in eukaryotes) are not known (some organelles come the closest but still fall short of comparable density), so it is, at least, less of a stretch to postulate that condition as a transient one. Secondly, I believe there is a very good reason why such an organism has never been discovered: it never existed because having those multiple (even if self-splicing) introns under the transcriptional-translational coupling mode of expression would be too much of a disadvantage. If so, one would think that such an organism would already have transcription and translation uncoupled. Again, we are unaware of such organisms – other than eukaryotes, of course. I think the hypothesis that we proposed with Bill Martin (ref. 59) and that I expand somewhat in the present paper offers a plausible chain of causation to tie it all together*.

Debate over the timing of the evolution of introns, and the relative contributions of intron gain and loss, is still raging, and, on balance, Koonin is perfectly entitled to assume the mitochondrial seed hypothesis as a starting point for discussions on the origin of spliceosomal introns, even if not everyone would agree. It is the second assumption with which I take issue.

The second assumption, again, is a case of assuming we know the ancestral state, and in this case concerns the nature of the endosymbiosis that gave rise to the eukaryote cell. This is the more difficult assumption to defend, and also that which is most important with regard to the novel ideas outlined in this paper.

Koonin's assumption is that the host of the endosymbiont that evolved into the mitochondrion is, as he puts it, a 'garden variety archaeon'. This is a commonly held assumption, but fails on closer inspection. I will not develop a full argument here, as I have recently submitted an article elsewhere on this (Poole & Penny, submitted), but here are two important issues. First, if archaea had already become a distinct domain prior to the origin of eukaryotes, eukaryote nuclear genes of archaeal origin should group specifically within the diversity of modern archaea, in exactly the same way as genes of mitochondrial origin fall within the diversity of modern bacteria, showing a specific relationship to alpha-proteobacteria. We see this clearly for mitochondrial-origin genes, but this has not been shown to be the case for supposedly archaeal-origin genes. That many archaeal genes are similar to eukaryotic genes supports a common origin for the two domains, but does not demonstrate that eukaryotes evolved from archaea. For this to be supported requires an explicit phylogenetic affinity between eukaryote genes and orthologous genes from a specific group of archaea, for instance methanogens.

*Author response: I maintain that the notion of an archaeal host for the mitochondrial endosymbiont remains the null hypothesis that we cannot reject. The argument that the 'archaeal' genes of eukaryotes should cluster within a specific branch of archaea is straightforward and substantial at first glance but fails upon closer consideration. Firstly, even with the mitochondria, the α-proteobacterial origin is demonstrable by phylogenetic trees for a frustratingly small number of genes [see Esser at al. (2004, Mol. Biol. Evol. 21: 1643–1660)]. It is, mostly, because some of these genes remained in the mitochondrial genome that we have no doubts as to the origin of the mitochondria. The genes that moved to the nucleus, in all likelihood, have experienced a substantial acceleration of evolution which complicates phylogenetic analysis. The same acceleration, probably, affected the host, archaeal proteins, hampering assignment to a specific group of archaea. On top of that, just as is the case with the mitochondria, we do not know the actual gene set of either the host of the endosymbiont, and given the amount of HGT in prokaryotes, this is far from being a moot issue. Some of the 'archaeal' proteins of eukaryotes do show phylogenetic affinity with a specific archaeal lineage, it is just that the signals are somewhat conflicting. Furthermore, the host did not even have to belong to one of the presently characterized groups of archaea. It very well could have been an archaeal branch that was outside the tree we are aware of and has either gone extinct or is still lurking somewhere (our understanding of archaeal diversity is very incomplete, indeed, more so than the understanding of bacterial diversity). It still would be an archaeon even if the word 'garden-variety' might be risky in such a case. In summary, the nature of the relationship between eukaryotes and archaea deserves further, careful investigation but at present I cannot see how we can reject the straightforward hypothesis of an archaeal host for the mitochondrial endosymbiont*.

The second criticism is that there are no known cases modern archaea housing bacterial endosymbionts. All endosymbioses that have generated organelles (chloroplast, secondary and tertiary endosymbioses) subsequent to the mitochondrion clearly involve eukaryotes (Archibald 2005 *IUBMB Life 57*:539), and modern examples of endosymbioses involving eukaryotes are widespread. While there is one example of a bacterium within a bacterium within a eukaryote (von Dohlen et al 2001 *Nature 412*:433) this is not equivalent to a bacterial-archaeal endosymbiosis. Consequently, the entire thesis rests on the unproven ability for archaea to be capable of hosting endosymbionts.

Author response: Yes, there is no direct evidence of bacterial-archaeal endosymbiosis. Neither is there any evidence of multiple instances of eukaryogenesis. I quite understand that this might make one feel uncomfortable but I think it is hard to doubt that here we are dealing with a rare event, apparently, an event that led to substantial evolutionary consequences just once in life's history. I think that the very grandiosity of the transformation of cellular organization that accompanied eukaryogenesis suggests that it could not be anything other than a near-unique occasion (more about that below with regard to Methanosarcina). This is not unlike the ultimate origin of cells: an enormously complex evolutionary transition for which we may not have a convincing scenario but we would not question the origin of cells from "some" kind of precellular (pre)life form because...what would be the alternative?

With this point in mind, I turn now to the speculative part of Koonin's paper, in which he argues that group II introns entered an archaeon via the alpha-proteobacterial endosymbiont. The consequence is an explosion in intron-numbers, compensatory evolution of a number of mechanisms of transcript quality control (the nucleus, Martin & Koonin op. cit.), nonsense-mediated decay (NMD), ubiquitinylation), and side-effects such as the evolution of linear chromosomes with telomeres and telomerase. The major point that needs to be discussed here is the expansion of group II introns, but before I come to that, I will deal with several minor points.

Koonin cites a paper wherein it was suggested that eukaryotic NMD evolved from a bacterial toxin-antitoxin system (Anantharaman & Aravind 2003 *Genome Biol 4*:R81), and extends this speculation by suggesting a mitochondrial origin for this toxin-antitoxin system. The original paper demonstrates similarity in so far as a universal domain, the PIN-domain, is found in both proteins involved in NMD. However, Koonin's hypothesis is phylogenetically testable – NMD genes which are homologous to bacterial genes should group with alpha-proteobacterial examples of these genes in gene trees. One can envisage complications, in that toxin-antitoxin systems are horizontally transferable, but without phylogenetic analysis the position Koonin takes cannot develop past the speculative stage. Again with ubiquitin, similarity to bacterial counterparts alone cannot establish the direction of evolution – more specific tests must be made if these suggestive links are to form the basis for hypotheses for the origin of key eukaryotic processes from bacterial or archaeal 'progenitors'. I am open to these possibilities, but feel it is insufficient to determine the direction of evolution based on similarity; this smacks of the Great Chain of Being, and we should be able to do better. One might equally argue that any such similarities are due to reductive evolution in bacteria and archaea (as I pointed out for group II introns above), but without more specific tests, the two opposing hypotheses are at loggerheads. We may not always be able to resolve the precise evolutionary history, but we can at least formulate alternative hypotheses and attempt formal tests.

*Author response: I am, of course, all for formal tests which are, however, beyond the scope of the present paper. I should add that such tests are quite hard in these cases, given the high level of divergence, in part, probably, due to accelerated evolution in eukaryotes. For small domains, like PINs and ubiquitin, this could be prohibitively difficult*.

Koonin's main argument however is that he can identify a selection pressure for the emergence of these mechanisms of quality control. Crucial to his hypothesis is the claim that group II introns, upon arrival in the archaeal host have, 'apparently, gone berserk within the host cell' (see also Martin & Koonin op. cit.). This is a key point, and one that the reader must accept in order to buy into any of the ensuing speculation. The argument builds upon the assertion that the small effective population size of this new 'prekaryotic chimera' precluded elimination of invading group II mobile elements by purifying selection. The host in this case is an archaeon (and thus asexual) with a small population size, but I am not sure we have any good examples to compare the model to.

Group II introns have recently been found in the archaea *Methanosarcina acetovorans *&*M. mazei *(Dai & Zimmerly 2003 *RNA 9*:14; Rest & Mindell 2003 *Mol Biol Evol 20*:1134), and seem to have become established as the result of horizontal gene transfer from bacteria (a recent transfer represents the ideal analogue to the 'primitive prekaryote' host genome). Here it seems that none of the group II introns are inserted in archaeal open-reading frames, and have a tendency to insert into the reverse transcriptase genes encoded by other group II introns, generating nested introns. I have no idea as to the effective population size of these two archaea, but that these elements have neither gone berserk, nor inserted into archaeal protein-coding genes does not serve to strengthen the model presented by Koonin.

*Author response: The case of Methanosarcina is, indeed, quite interesting. Surely, their genomes are full of acquired bacterial genes, and there are some Group II introns as well but all this has not made them eukaryotes. On the one hand, that does not strengthen the model I discuss in this paper...but, I do not think this weakens it either. What this case does confirm, is that eukaryogenesis is no small feat (see above) – in a way, one might be tempted to think of Methanosarcina as a failed eukaryote although we have no idea whether or not some sort of transient endosymbiosis was involved*.

Examples of asexual lineages with small population size, such as *Buchnera *are more clearly analogous to the endosymbiont, not the host, and here it seems that explosive expansion of existing selfish elements is not a feature of these lineages; the opposite is true. Population size could well play a role, and is in principle testable, but to my knowledge the only examples of massive expansion of selfish elements are in organisms with meiotic sex. Here it has been argued that selfish elements expand at the expense of the host because outcrossing permits spread, provided that the cost to the host is less than 0.5 (Hickey 1982 *Genetics 101*:519). Species with smaller effective population sizes do seem to have a higher density of elements (e.g. complex multicellular organisms), but these are all sexual. Conversely, in asexual systems, the theoretical expectation is that selfish elements should be rapidly eliminated, because they impart a selective disadvantage on the host, meaning that element-free lineages will outcompete those with elements (Hickey op. cit.; Johnson & Brookfield 2002 *J Evolution Biol 15*:42).

Under Koonin's scenario, mitochondrial group II introns inserted into the host genome sweep to fixation in the population via drift, and subsequently expand in numbers. It is of course not impossible for drift to sweep a selfish element to fixation, even under Hickey's model. However, should these then expand significantly in number over a longer evolutionary period? One key point that emerges from Hickey's original treatment of this problem is that there should be attenuation of selfish element activity because in an asexually reproducing lineage the reduction in fitness imparted on the host by a given element is also the fitness reduction on the element. There is therefore selection on the element to become less virulent. This is exactly the opposite of what Koonin's model (and indeed Martin and Koonin's model) requires.

It has recently been argued that element overload is one probable cause of extinction of obligately asexual lineages that have evolved from sexual lineages (Arkhipova & Meselson 2005 *Bioessays 27*:76). So, in my mind, for Koonin's 'berserk introns' theory to work over a long enough time scale for several complex systems of quality control to emerge, I would argue that he should at least have invoked the emergence of facultative meiotic sex. The problem with this is that sex, with its two-fold reproductive cost, must be invoked under a scenario where there is a population of primed selfish elements 'waiting' to spread. While the level of sexuality can be increased in a facultatively sexual population, this is not so for an asexual population (Johnson & Brookfield op. cit.). As the ever-present difficulty with models for the origin of sex is accounting for the short-term selective advantage for sex, this would represent a rather backwards way of approaching the problem!

A further point worth considering is that Koonin's archaeon model weakens the introns-late stance because it eliminates the possibility of the mitochondrion entering an early eukaryote stem lineage that had already evolved meiosis (see Ramesh et al 2005 *Curr Biol 15*:185) for recent discussion on the timing of the evolution of meiosis). One has to accept the necessity of stem-lineage eukaryote ancestors irrespective of the absence of extant primitively amitochondriate eukaryotes. (By stem lineage I do not mean extant eukaryotes that were designated archezoa. I mean lineages of eukaryotes that have gone extinct and which diverged from the lineage leading to the Last Eukaryotic Common Ancestor – see Donoghue 2005 *Paleobiology 31*:553 for standard definitions of stem and crown groups) This is because, even if, as Koonin argues, the ancestors of eukaryotes were archaea bearing alpha-proteobacterial endosymbionts, there are no intermediate stages between this hypothetical ancestor and modern eukaryotes (Poole & Penny, submitted). By invoking a sexual, eukaryotic host, the spread of introns in eukaryotes can better be accounted for under our current understanding of selfish element spread in sexual populations.

*Author response: I appreciate these thoughtful comments. Admittedly, the connection between sex and propagation of selfish elements during eukaryogenesis has not been explored in sufficient detail. The "necessity of stem-lineage eukaryote ancestors", though, is very, very hard to accept, and if we do, we will also have to accept that we have no clue whatsoever as to how, under what sort of selective pressure they would evolve the complex features of the eukaryotic cell. Obviously, I believe that our model with Bill Martin (Ref. 59), which I extend a little here, goes some way to propose a plausible chain of causation for eukaryogenesis (see *Fig. 2*in the present paper) without invoking mysterious, extinct creatures. This does not mean such creatures have never existed, just that, following Occam razor, we probably should try to explain eukaryogenesis as best we can without their help*.

In summary, I am not convinced that the host in the endosymbiosis leading to modern eukaryotes was an archaeon. This should be detectable phylogenetically, picking out a specific group of archaea as the closest relatives of eukaryotes in exactly the same way as this is possible for the mitochondrion. Second, no archaea have been identified which carry endosymbiont bacteria, so accepting Koonin's assumption would require that all extant archaea have subsequently lost this capacity. Nor am I convinced that the transfer of group II introns into an archaeal host from its bacterial endosymbiont would have led to massive expansion of group II elements. This does not seem to fit with our current knowledge of selfish element spread under an asexual reproductive mode. That modern methanogenic archaea of the genus *Methanosarcina *have not suffered from massive intron expansion, seems to confirm this suspicion.
